# The systematic cultural adaptation of a UK public health cancer awareness raising programme for Malaysia: the Be Cancer Alert Campaign

**DOI:** 10.1093/tbm/ibz134

**Published:** 2019-10-04

**Authors:** Désirée Schliemann, Tin Tin Su, Darishiani Paramasivam, Saunthari Somasundaram, Nor Saleha Binti Ibrahim Tamin, Maznah Dahlui, Siew Yim Loh, Michael Donnelly

**Affiliations:** 1 Centre for Public Health and UKCRC Centre of Excellence for Public Health, Queen’s University Belfast, Belfast, UK; 2 Centre for Population Health (CePH), Department of Social and Preventive Medicine, University of Malaya, Kuala Lumpur, Malaysia; 3 National Cancer Society Malaysia, Kuala Lumpur, Malaysia; 4 Ministry of Health, Kuala Lumpur, Malaysia; 5 Department of Rehabilitation Medicine, Faculty of Medicine, University of Malaya, Kuala Lumpur, Malaysia; 6 South East Asia Community Observatory (SEACO), Monash University Malaysia, Bandar Sunway, Malaysia; 7 Facultas Public Health, University Airlangga, Surabaya, Indonesia

**Keywords:** Cultural adaptation, Mass media, Breast cancer, Colorectal cancer, Interdisciplinary research, Malaysia

## Abstract

Increasingly, policy and research attention is being directed toward improving global health in low- and middle-income countries. This study investigated the cultural adaptation of a UK-designed and developed evidence-based mass media campaign with the aim of improving colorectal cancer and breast cancer awareness in Malaysia. Guided by the heuristic framework of cultural adaptation, a multidisciplinary team adapted the UK Be Cancer Aware programme for implementation in the Malaysian context. The approach included five steps: (a) information gathering and needs assessment; (b) preliminary design; (c) preliminary testing; (d) refinement; and (e) final trial. Key findings from the information gathering stage related to the need to take into account differences in ethnicity, religion, and beliefs about cancer. Discussions with experts indicated that particular words were not acceptable in Malay culture and that specific aspects were “taboo” (e.g., showing pictures of breasts in relation to breast cancer on TV). Stage 3 of the analysis revealed that the presentation of cancer survivors rather than health professionals on programme materials was preferred by Malaysians and that there was a poor level of awareness about colorectal cancer. The results were used systematically to adapt two culturally suitable cancer awareness mass media campaigns for implementation in Malaysia. The developed materials were in line with government priorities and took into account the local health care system structure. The establishment of a partnership with key stakeholders (e.g., the Ministry of Health and the lead patient advocacy organization) and the application of a systematic approach to address cultural factors and resource constraints contribute to the successful implementation of public health programmes in global health settings.

## Teaser

We used a cultural adaptation framework and local partnership working in Malaysia to translate and implement an established cancer awareness-raising mass media campaign.

**Implications**

**Practice:** An evidence-based culturally acceptable cancer mass media campaign is available, which may be adapted to assist efforts in other countries and contexts to improve health behaviors.

**Policy:** Adaptation of an evidence-based programme facilitates the time-efficient implementation and delivery of urgently needed public health interventions in resource-poor settings.

**Research:** Cultural appreciation and adaptation is a key investigative activity in the successful implementation of public health programmes and should follow rigorous implementation and evaluation processes.

## INTRODUCTION

Cultural adaptation is an important process in implementation science in recognition of the often neglected role of context and, so, the process focuses explicitly on the cultural context of translating an intervention for delivery and uptake by a new target population [1]. In essence, this process involves altering and testing systematically an evidence-based intervention (EBI) that has been proven to be successful for one population to make it culturally appropriate and relevant for a different population [1,2]. It is important to acknowledge that culture is not a fixed set of characteristics limited to race and ethnicity but, rather, a constantly evolving and dynamic concept which encompasses the collective views, beliefs, norms expectations, traditions, customs, and interactions that distinguish population groups [1,3,4]. The first framework to guide this process by Barrera and Castro [5] and similar frameworks since [6,7] have been reviewed extensively across different health care disciplines and settings [8–11]. Importantly, empirical studies indicate that a culturally adapted EBI is similarly effective to its original version [4,12]. Recently, the various frameworks were used to develop a consensus approach to the stages of cultural adaptation by Barrera et al. [13].

The main benefits of cultural adaptation are the increased chance of interventions being successful and the saving of resources (e.g., time, costs, and staff) to support intervention design. Therefore, countries with a lack of resources for preventative and diagnostic services (i.e., low- and middle-income countries [LMIC]) may particularly benefit from evidence-based interventions developed in high-income countries with a greater budget allocated to health care and preventative services [14]. Mindful of these benefits, collaborators from Queen’s University Belfast (QUB) and the University of Malaya (UM) identified the Be Cancer Aware Campaign (BCA) run by the Public Health Agency Northern Ireland (PHA NI) that was informed by the Be Clear on Cancer (BCOC) campaign in England as successful mass media campaigns to improve awareness about different types of cancer in NI. Findings from these campaigns reported increased awareness about the signs and symptoms of cancer [15] (Public Health Agency. Evaluation Report Breast Campaign; Evaluation Report Primer and Lung Campaign). Furthermore, patients who attended general practitioner practices reported key campaign-related symptoms correctly [16] and appropriate referrals of patients with suspected cancer increased in the implementation areas. Also, an increase in diagnostic investigation activity and a shift toward earlier stage diagnosis was reported [17]. Breast cancer (BC) and colorectal cancer (CRC) were the two commonest cancers in Malaysia in 2011 (i.e., 17.7% and 13.2% of all cancer cases, respectively) [18]. Stage of cancer detection in Asia, including Malaysia, is late compared to western countries, and it is estimated that up to 50% of premature cancer deaths could be prevented if diagnosed and treated early [19]. One of the main barriers toward early diagnosis is the lack of awareness of signs and symptoms as well as barriers toward cancer screening, such as negative perceptions, fear, denial, and trust in complementary alternative medicine [20,21]. However, evidence about the effectiveness of mass media cancer awareness campaigns to improve cancer awareness and beliefs in South East Asia is lacking [22]. Therefore, the objective of this research was to describe the systematic adaptation of the BCA for BC and CRC to suit the Malaysian culture. 

### Cultural adaptation: methods and findings

The study has been reported in line with template for intervention description and replication guidelines [23] ((abcdefghlink)Supplementary Material 1(abcdefghxref)). This section describes the conduct of the cultural adaptation of the BCA campaign to suit the context of Malaysia and is the first step in the development of the Be Cancer Alert Campaign (BCAC) as outlined in the previously published study protocol [24]. Barrera and Castro’s heuristic framework of cultural adaptation was used to guide the cultural adaptation process. The stages of the process are based on a consensus that emerged from various adaptation frameworks and guidelines [5,13]. In line with implementation science guidelines for global health practice in terms of involving policy makers and other stakeholders [14], the panel that advised the adaptation process comprised experts from the UK (i.e., QUB and the PHA NI) and Malaysia [i.e., UM, National Cancer Society Malaysia (NCSM) and Ministry of Health Malaysia (MOH)].

### Stage 1: information gathering and needs assessment

#### Methods

Researchers from QUB and UM met with programme coordinators from the PHA NI in June 2016 to gather information (including all campaign materials and internal PHA NI evaluation reports) to learn about the development, implementation, and evaluation of the BCA campaign (PHA NI. Evaluation Report Breast Campaign; Evaluation Report Primer and Lung Campaign). Panel members from both countries co-conducted research workshops in order to understand the local context and to identify similarities and differences between the two countries, particularly regarding the population and health care system. Furthermore, we conducted two systematic literature reviews to gain a better understanding of the cancer landscape in Malaysia and to identify gaps in cancer knowledge and the health education needs of Malaysians and how to address them. The first systematic review aimed to identify the level of BC and CRC awareness in Malaysians as well as barriers towards screening [25]. The objective of the second systematic review was to examine the effectiveness of mass and small media campaigns to improve cancer awareness and screening rates in Asia [22]. This process helped the research team to identify mismatches between the different target populations. The evidence from the systematic review activity contributed to the cultural adaptation process by, for example, facilitating the identification of similarities and differences between populations and providing a research-informed rationale for amendments to the intervention/campaign.

#### Findings

The differences and similarities between the two countries that were identified by the panel members are outlined in Table 1. Obvious differences between the two populations are the main ethnicities, languages, and religions practiced. Malaysia is a diverse country with Malays and other indigenous communities comprising the majority of the population (69.1%), followed by Chinese and Indians (23.0% and 6.9%, respectively) [26]. Religion plays a dominant role in Malaysia—Islam is practiced most commonly (61.3%), especially among Malays, followed by Buddhism (19.8%), Christianity (9.2%), and Hinduism (6.3%) [27]. Studies of South East Asian populations that explored the role of religion and cultural beliefs in cancer prevention and early detection suggested that social stigma and fear about cancer were barriers to earlier detection [28,29]. A study by Daher et al. reported that women in the Middle East, where Islam is the most dominant religion (similar to Malaysia), were highly reluctant to discuss breast or gynaecological-related issues with their doctors and even more reluctant to undergo a physical examination [30]. Also, studies of South East Asian women as well as Muslim immigrant women in the USA reported that the concept of preventative screening is novel to women and that illness is seen as fate [28,31] and as punishment from god [32]. In addition, according to socio-cultural norms, often, Muslim women are expected to put the health of their families before their own health [31]. Whilst religion and cultural norms appear to be barriers toward early detection of cancer, a meta-analysis reported an association between spirituality and/or religion and better-reported physical health in cancer patients [33]. In brief, best available evidence and local discussions indicated that religion should be taken into account in the development of cancer-related interventions; and practitioners and researchers should focus on elucidating the importance of cancer prevention.

**Table 1:** Description of the target populations (Northern Ireland and Malaysia)

	Be Cancer Aware	Be Cancer Alert	Explanation/justification/challenges/consideration
Setting			
Country	Northern Ireland	Malaysia Study area: Federal Territory of Kuala Lumpur and Selangor State	Different national TV channels, radio stations, and print advertising mediums required
Population statistics	1.88 million	32.4 million [43] Study area: ~200,000	Although the study population is smaller in the selected study area in Malaysia (Petaling Jaya) than in Northern Ireland, population density is higher in the selected study area.
Socioeconomic status [44]	High-income country	Upper middle-income country	Different resources are available for prevention and early diagnostic services.
Health Care System	National Health Care System (NHS)	Dual-tier Health Care System (82% of inpatient care and 35% of ambulatory care covered by public sector)	Cost of cancer treatment is of greater concern for Malaysians as treatment is paid for UK citizens by the NHS (not all cancer treatment costs are covered in Malaysia).
Study population			
Age	Across all ages with primary target group aged 50 years and above	Aged 40 years and above	Since the target population was males and females aged 40 years and above, we only recruited from that age category.
Ethnicity (most common)	Northern Irish	Malaysian (Malay/Chinese/Indian)	Needs to be taken into account when creating visual materials. Stigmas, beliefs, and role of family likely to differ between countries.
Language	English	Malay, English, Cantonese/Mandarin/Hokkien, Tamil	Although Malay is the official language in Malaysia, some families and communities only communicate in English/Tamil/Chinese and are not comfortable/able to communicate in other languages.
Religion	Christianity (Catholic, Protestant)	Islam, Buddhism, Christianity, Hinduism	Religion is likely to influence some people’s beliefs and health behaviors.
Socioeconomic status	All socioeconomic groups	All socioeconomic groups	Needs to be considered in terms of advertisement channels (location, stations, etc.) and use of language
Rural/urban	Rural, semiurban, and urban communities	Semiurban and urban communities	Consider for advertisement placement, for example, TV covers rural population as well but print advertisement is likely less cost effective
Cancer risk^a^			
Incidence rate (percentage of all cancer cases/crude rate/age-standardized rate per 100,000)	Breast cancer: 22.3/148.4/10.3 Colorectal cancer (male): 9.5/71.7/93.8 (female): 8.5/56.5/62.5	Breast cancer: 17.5/28.6/31.1 Colorectal cancer (male): 16.3/11.7/14.6 (female): 10.7/9.5/11.1	Breast and colorectal cancer present a significant burden for the Northern Ireland and Malaysian population and health care system.
Cancer mortality (crude rate/world age-standardized rate per 100,000)	Breast cancer: 33.0/15.9 Colorectal cancer (combined): 23.8/10.7	Breast cancer: 18.7/ 18.4 Colorectal cancer: 10.7/ 11.2	Cancer mortality compared to incidence is higher in Malaysia (i.e. more people are likely to die from cancer in Malaysia)
Stage at detection (incidence %)	Breast cancer: Stage 1: 42.4%; Stage 2: 39.7%; Stage 3: 12.8%; 5.4%; Unknown: 5.5% Colorectal cancer (combined): Stage 1: 20.0%; Stage 2: 28.6%; Stage 3: 29.2 %; Stage 4: 22.2%; Unknown: 10.2%	Breast cancer: Stage 1: 20%; Stage 2: 37%; Stage 3: 23%; Stage 4: 20% Colorectal cancer: Stage 1: 10%; Stage 2: 24–25%; Stage 3: 30%–32%; Stage 4: 33%–36%	Breast and colorectal cancer are detected at later stages in Malaysia compared to the Northern Ireland.
Age-specific incidence rate	Breast cancer: most cancer cases presented in women aged over 50 years Colorectal cancer: most cancer cases presented after the age of 65 years	Breast cancer: most cancer cases presented in women aged over 45 years Colorectal cancer: most cases presented after the age of 60 years	Breast and colorectal cancer occur earlier in males and females in Malaysia compared to Northern Ireland.
Screening guidelines			
Breast cancer	Women aged between 50 and 70 years are invited for a mammogram every 3 years (population-based screening).	(a) Women aged 40 and above are recommended to undergo a mammogram if at high risk for breast cancer (opportunistic screening). (b) Women aged between 50 and 74 years are recommended to undergo a mammogram every 2 years (opportunistic screening) [45].	Screening attendance in the UK is higher already due to regular screening invitations, and citizens are more likely to be familiar with the condition as well as screening.
Colorectal cancer	Men and women aged between 60 and 74 years are invited for a fecal occult blood test every 2 years (population-based screening).	Men and women between 50 and 75 years and above should conduct a yearly fecal occult blood test (opportunistic screening) [46].	

^a^Reported for both sexes for CRC and females only for BC; statistics for Northern Ireland are obtained from the Northern Ireland Cancer Registry report 2013–2017 [47] and statistics for Malaysia were obtained from the Malaysian National Cancer Registry report 2007–2011 [18] and GLOBOCAN 2018 [48].

In Malaysia, Bahasa Melayu is the official language and most Malaysians speak English. Each ethnic group also practices their own language (Tamil, Mandarin, Cantonese, and Hokkien). The main economic differences between the UK and Malaysia are that Malaysia is a middle-income country, whereas the UK is a high-income country [34]. The health care system in Malaysia is dual tiered with well-established and used public and private health care services [35]. The public health services are highly subsidized, that is, patients pay RM1 (equivalent of 20 cents) for all outpatient treatments and RM5 for specialist visits, but there are out of pocket expenses such as transport and food for inpatient stays. In the UK, the National Health Care System covers cancer-related services and treatments free at the point of delivery. Financial concerns regarding cancer treatment are more prevalent in Malaysia—qualitative interviews indicated that patients were fearful about not being able to afford cancer screening or treatment [21,29,36]. Economic hardship may affect a patient’s family in Malaysia, whereas welfare benefits are available in the UK (unlike Malaysia) as a means of providing some support to cancer patients and their families [37]. In Malaysia, BC and CRC are detected at a later stage and cancer mortality in relation to cancer incidence is greater compared to NI (Table 1), thereby highlighting the urgent need for advances in early cancer detection in Malaysia.

Findings from the first systematic review regarding cancer awareness highlighted that Malaysians recognize “lump in breast or under armpit” and “breast pain” as the most common signs for BC; and rectal bleeding, blood in stool, and changes in bowel habits as the top-noted signs for CRC. Furthermore, barriers toward BC screening were identified as pain and lack of time, whereas barriers toward CRC screening were found to be embarrassment, fear of discomfort, and uncertainty about whether or not people should take a screening test. The second systematic review identified that screening campaigns in Asia tend to be conducted through small media communication and information about the evaluation of mass media campaigns is lacking [20]. There was only one mass media campaign in Malaysia and it focused on oral cancer awareness [38]. Loh et al. also highlighted that there are numerous cancer awareness raising campaigns in Malaysia delivered by industry and NGOs, in particular BC awareness campaigns. However, scientific evaluation of these campaigns is lacking [39].

### Stage 2: preliminary design

#### Methods

Two expert panel discussions (EPD) were conducted in August 2017 with the objective of investigating the appropriateness and cultural acceptability of the BCA campaign materials: that is, key messages, print materials, and radio and TV scripts for use in Malaysia. We invited experts from the following areas: family physicians (*n* = 3), public health (*n* = 4), language and linguistic (*n* = 1), cancer advocates (*n* = 4), and media and communication (*n* = 5). The panel comprised fluent native speakers of the four main languages (Malay, English, Cantonese, and Tamil). Each EPD took one working day and was guided by one of the lead investigators (TTS). The format of the EPD was structured, that is, direct questions were asked regarding the clarity and acceptability of each type of campaign material and about suggestions for further improvement. The discussions were tape-recorded and then destroyed after transcription of the EPD.

#### Findings

The expert panel deemed that the cultural relevance of the materials and key messages therein needed to be modified (Table 2). Words that may not be easily understood or misinterpreted needed to be changed and cancer statistics and health system-related terminology needed to be adapted to be culturally appropriate for Malaysians. For example, “general practitioners” in Malaysia only refers to doctors working in private clinics and, therefore, needed to be changed to “doctor.”

**Table 2:** Findings and quotes from the expert panel discussion

Colorectal cancer Key messages: • “GP” refers to private health care only in Malaysia and needs to be changed to “doctor” “I think better to change the GP to a Doctor.” • “Poo” is not commonly used in Malaysia and needs to be changed to “stool.” “I’m not prefer to use ‘poo’ because this word widely referred or used by kids, Prefer to change ‘poo’ to ‘stool’.” • Malaysians should visit a doctor sooner than after 6 weeks. • Survival statistics are different in Malaysia. • Strengthen message by saying “cancer can be cured if found and treated early.” “I think using the word cured will be more catchy and attract more attention.” • Message “listen to your body” is too soft and does not call for action. “I think listen to body is too weak.” “Listen to body, bodies have many parts, which part are we referring to?” Radio advertisement • 40 s are too long (change to 30 s) “I think better to have a 30 sec advertisement.” • Adapt concept to local culture. • Messages should be more direct and sentences need to be shorter. “Better get straight to the point and easy understand.” • Malaysian’s may not understand “number two.” “Basically, Malaysian won’t use the ‘number two’ instead use a long call or nature’s call.” TV advertisement • Concerns about cultural acceptability in Malaysia to show someone sitting on the toilet. “I do not think so it will be culturally acceptable to show someone sitting in the toilet.” • Change ethnicity of person in the TV ad. “Prefer to have local ethnicity person to be in the TV advertisement.” • Make concept more culturally acceptable. Print advertisement • Too wordy; less text preferred. “Malaysian won’t read too long sentences, better change to local a local flavour.” • Make poster culturally suitable (i.e., change ethnicity of person delivering the message). “This poster is more on UK culture and it’s better to change according to Malaysian context.”	Breast cancer Key messages: • Change symptom terminology so that it is easily understood by Malaysians. • Change “GP” to “doctor.” “Must improve on the GP words to doctor or healthcare provider.” • Check statistics are in line with Malaysian survival rates. “9 out of 10 women survived, I wonder where the data are from. Is it local data or their data?” • Change “listen to your body” to “check your breasts” for a more direct message. • Change “caught” to “detected” or “found” as it can be misleading when translated. • Convey a positive message of cure. “Cancer is known as a death sentence. So, I think key messages have to be positive, only positive messages.” • Have a call to action. Radio advertisement • Use local people to speak to be in line with local tone and to improve clarity. “Try to change the tone or voice of advertisement presenter to attract more attention.” • Shorten ad. • Record in different languages to reach different population groups. TV advertisement • Work around cultural taboos, that is, cannot say “nipple” on TV or “breast” except when talking about “breast cancer.” • Taboo to show breasts on TV. “I do not think can advertise the breast in the mainstream TV channel like TV3.” • Use ordinary people in ordinary environment for people to be able to relate. “Try to have an ordinary person, who doesn’t look rich, who doesn’t look famous.” • Bring in family component as it is important in Malaysian culture. “Add their family around, with average house setting.” • Tone should be encouraging. • Choose TV channels commonly watched during daytime by housewives. “As the target groups are including housewives, choose to advertise during ladies talk show.” Print advertisement • Needs to be appealing to all three races. “It would be good to having three survivors by three different races standing together.” • Visuals need to be more interesting/appealing. • Shorten the message. • Make it locally relevant.

*GP* general practitioner

Overall, the experts judged that more direct messages and words of “action” were required (e.g., change “listen to your body” to “check your breasts”). A series of 30 s radio spots were thought to be sufficient to communicate the key messages. In particular, the BC TV scenarios needed to be changed as it was not culturally acceptable to demonstrate BC symptoms on mannequins or real people, particularly with respect to the Muslim community. A suggestion from the panel to use Malaysian celebrities was overruled in favor of presenting ordinary people in everyday environments and highlighting the importance of family. Regarding posters, findings from the EPD suggested that “visuals” needed to be inclusive to appeal to all three main ethnicities in Malaysia.

### Stage 3: preliminary testing

#### Methods

Six structured group discussions (GD) were conducted from August to October 2017 with participants reflecting the target population, that is, Malaysians aged 40 years and above. Two GD were conducted with each ethnic group (Malays, Chinese, and Indians), one GD with participants from low-income backgrounds and one GD with participants from middle-income backgrounds. We excluded participants who were health care professionals, given that the aim was to target the media materials toward the general (lay) population. Each GD included 10 participants (5 males and 5 females) and took approximately 1 hr. The aim was to (a) identify participants’ views about the key messages and materials from the BCA campaign and to (b) identify the most commonly used media channels by the target population. A researcher who speaks Malay, Tamil, and English fluently (DP) conducted and recorded the GDs in Tamil or English with Indian participants and in English or Malay with Chinese and Malay participants. Materials from the BCA campaign were presented and discussed at each GD. The content of each GD was transcribed by two professional transcribers who were proficient in the various languages and then translated into English by a professional translator. Analysis of the interviews was conducted by two researchers (DS, DP) independently and findings were discussed and summarized.

#### Findings

Findings from the GD are summarized in Table 3. Similar to the EPD, participants expressed the view that the three ethnicities should be represented in the materials. Furthermore, participants reported that it would be easier to understand the messages if they were delivered in their respective local language. Participants from high- and low-income backgrounds did not find the words “nagging” and “dimpling” easy to understand and, so, these terms were amended. The general consensus was that the presentation of facts and numbers such as “9 out of 10 women” should be retained if they had scientific support. Views about the general tone of the TV advertisements were mixed. Some participants reported that presenting TV material in a way that frightened people would be an effective method, whereas other participants thought that TV messages should be encouraging in order to empower people to visit a doctor if they noticed any abnormal changes. Therefore, a consensus emerged that indicated that cancer survivors instead of doctors should deliver the messages in all communication channels. Furthermore, participants agreed that the inclusion of celebrities was likely to attract attention to the BC campaign. However, celebrities alone might send out the wrong message, that is, it may indirectly imply that “ordinary” women cannot afford treatment and only rich people can survive cancer. According to participants, there was too much text on posters and radio and TV advertisements needed to be shortened. There was a low level of familiarity with the colon, which confirmed the need to raise awareness about CRC signs and symptoms. Therefore, it was suggested to include an image of the colon on print advertisements to improve understanding. Participants also highlighted that they did not tend to check their stool.

**Table 3:** Findings from group discussions

	Suggestions	Quote
Colorectal cancer	• Difficulty in understanding what colorectal cancer is • Include diagram/picture of colon to aid understanding	“Usus (colon) means? Usus (colon) is what?” *Chinese, Female, Low Income* If there is an image that is used. […] Do you have a poster to help people understand colorectal? *Chinese, Male, Low Income*
	• Difficult word: “nagging”	“What do you mean by nagging?” *Chinese & Indian, High Income*
	• People usually do not look at their stool	“We just do our business and flush, we do not really look at it.” *Chinese, Male High Income*
Breast cancer	• It is not acceptable to show breasts (real or mannequin) on TV or street advertisement)	“If it’s an internal organ it’s still OK. But externally, still not OK [to show a picture highlighting breast cancer symptoms], for public view quite difficult.” *Malay, Male, High Income*
	• Messages including numbers, e.g. “9 out of 10 women can survive cancer” are catchy	“You said that 9 out of 10 women survive breast cancer if found and treated early. That is to catch the attention. I think that one must say first, in the beginning.” *Indian, Female, High Income*
General suggestions	• Cancer survivor is preferred over doctor to communicate message/ share story • Celebrities would also attract attention • Real person is preferred over cartoon/animation	“It will be better to have cancer survivors talk. Doctor may be ok as well but not any random people without the cancer, or related to the cancer. Not just for acting sake by the artist. People will know it’s fake. It’s better to take real patient.” *Malay, Male, High Income* Cartoons are inappropriate, not serious. *Malay, Female, Low Income*
	• Shorten messages on poster	“When you put too many wordings, people won’t be stopping and reading it patiently. [...] Make it short lah.” *Indian, Male, High Income*
	• Messages should be delivered by different ethnicities in local languages, that is, multicultural (Malay, Chinese, and Tamil)	“Another one I see, for TV ads, mix Chinese, Indian, Malay, combine together become 1 Malaysia, with 1 Malaysia message. One against cancer […].” *Malay, Male, Low Income*
	• Highlight urgency of the message to undergo screening	“Check early is very important, that’s what you tell people.” *Chinese, Female, High Income*
	• Different opinions on whether to have a hopeful and encouraging or scary tone to the messages	“We Malaysians, if not scared we won’t take action. […] Maybe a guy crying at the graveyard […] and he notices blood on his attire.” *Malay, Female, High Income* “I think your poster should be giving hope and encouragement to go to [see a doctor].” *Chinese, Male, Low Income*

The most popular media channels amongst Malays were TV 3, Astro, Sinar FM, and Hitz FM. Chinese watched mainly 8TV and listened to Lite FM and Mix FM. Indians reported watching TV3 as well as Vijay TV and listening to Thr Raaga or Minnal FM. Most participants did not read newspapers or magazines. The most commonly mentioned newspapers were Berita Harian, Harian Metro, Utusan Malaysia, The Sun, or Star. Most participants used social media, particularly Facebook and YouTube, and it was suggested that these channels should be used for campaign purposes.

### Stage 4: refinement

#### Methods

The research team collaborated fully in all stages of the adaption process and the research project generally. Working collectively, the team analyzed the data that were gathered from the first three stages of cultural adaptation and, then, devised suitable messages for the Malaysian awareness campaigns. Print advertisements, Radio, and TV scripts were developed together—local designers were guided by the identified key messages and findings from the needs assessment between November 2017 and January 2018. Language experts forward- and back-translated all materials to ensure linguistic accuracy. Media communication channels were chosen in discussion with media experts and based on findings from the BCA campaign and the feedback from the GD.

#### Findings

The core concepts of the BCA messages were kept and only the wording was changed to be more culturally relevant and acceptable to Malaysians (Table 4). The media campaign strategy was designed in line with popular media channels identified through the EPD and GD and in discussion with media experts. Furthermore, learning points from the BCA were taken on board, that is, TV and clinic posters were strong communication mediums to reach the target population.

**Table 4:** Adapted key messages for the Be Cancer Alert Campaign

Be Cancer Aware	Be Cancer Alert	Explanation/justification
Colorectal cancer		
If you’ve noticed a change when you go to the loo, like blood in your poo or looser poos for six weeks or more, see your GP.	If there is blood in your stool and you are experiencing constipation or diarrhea for several weeks, see a doctor urgently.	Words such as “loo” and “poo” are not commonly used in Malaysia and need to be changed. Symptoms need to be made clearer.
It could be nothing. But you won’t know until you let your GP check.	The changes in your bowel habits could be nothing. But you won’t know until you let your doctor check.	This message needs to be clearer. “GP” needs to be changed to “doctor.”
9 out of 10 people survive bowel cancer when it’s found early.	Colorectal cancer can be cured if it is found and treated early. Colorectal cancer is the second most common cancer in Malaysia.	Survival statistics are not so clear for Malaysia. However, statistics are powerful and may help to convey the message.
Listen to your body and talk to your GP.	Watch out for the cancer signs, see the doctor and get checked.	Be more specific, the message is too soft. Change GP to doctor.
Breast cancer		
Lumps aren’t the only sign of breast cancer. If you notice any changes to your skin such as dimpling or nipple changes (turned in, a discharge, crusted), see your doctor straight away.	Lumps aren’t the only sign of breast cancer. If you notice any unusual changes to your skin or nipple see your doctor straight away.	We cannot use the word “nipple” on TV. Therefore, the TV advertisement, we use the statement “Lumps aren’t the only sign of breast cancer” at the end of the advertisement as, however, for the brochures and posters, the message remained similar. Words that were difficult to understand by the public (dimpling, crusted were removed)
9 out of 10 women survive breast cancer when it is caught and treated early.	9 out of 10 women survive breast cancer if it is found and treated early.	Statistics are powerful and “catchy” and may help to convey the message. Malaysian statistics are similar to statistics in the UK. “Caught” is understood differently in Malaysia and needed to be changed to “found.”
Listen to your body and talk to your GP.	Check your body and see the doctor.	Be more specific, the message is too soft. Change GP to “doctor.”

The print materials are highlighted in Supplementary Material 2. For the CRC campaign, the team decided to feature the colon as many people were not aware of what CRC was. Furthermore, the headline “Don’t be shy to check your stool” was chosen to make people aware of some of the actions that can be taken to check for CRC symptoms. The BC visuals feature three female BC survivors, representing each ethnicity, and a Malaysian supermodel (celebrity) to attract further attention to the campaign. Cancer survivors were aged above 40 years to reflect the target population. This is in line with the health belief model that guided the design of the campaign, that is, the target population needs to be able to identify their susceptibility to the disease in order to increase the likelihood of appropriate action.

The CRC and BC TV videos featured a local survivor (male for CRC and female for BC). The scripts focused on presenting the key messages (cancer awareness and importance of early detection). The TV campaign designers advised that it would not be acceptable for breasts (real or in the form of mannequins) to be shown on TV and that the words “nipple” or “breast” (except for “breast cancer”) were taboo on TV. We chose two Malay (TV3 and TV9) and one Chinese (8TV) TV station to air the BCAC advertisement based on the GD feedback and to reach the two largest ethnic groups in Malaysia. Furthermore, Indians also understand and commonly watch Malay channels. The same videos with voiceovers for the various languages were presented on each station.

The radio advertisement, too, was presented from the view of two cancer survivors (one male and one female for CRC to raise awareness that it affects both genders; two females for BC). The radio scripts focused on the key symptoms as well as the importance of early detection since increasing awareness about signs and symptoms of CRC and BC was the main objective of the campaigns. Feedback from the EPD suggested shortening the advertisement. Hence, each radio advertisement lasted only 30 s compared to 40 s in the BCA campaign. The TV advertisement targeted mainly Malays and Chinese. We created English and Tamil radio advertisements, which were aired on Lite FM and Thr Raaga to reach all Malaysians listening to English radio stations and reach Indians who may not watch the chosen TV channels.

In addition, we developed a social media campaign, which was distributed through the National Cancer Society Malaysia (NCSM) Facebook page. Facebook was chosen as it is the social media platform most commonly used by the target population (as identified in the GD). Similar to the BC TV advertisement, the designers of the social media materials advised that Facebook will block materials that display nudity. Therefore, presentation of the materials about the signs and symptoms of breast cancer were disguised and subtle. Finally, we developed a website that highlighted the campaigns and contained all the campaign information and materials. In addition, NCSM promoted a toll-free helpline on campaign materials, which offered professional advice regarding cancer-related topics.

GD identified that few people read newspapers or magazines. Therefore, we decided not to invest resources in newspaper articles given the limited budget for the campaign, which was funded mainly via the research grant. However, we organized a media launch which attracted journalists who wrote about the campaigns.

### Stage 5: final trial

#### Methods

In February 2018, the final versions of the various materials were vetted and approved for appropriateness and cultural suitability by the following stakeholders and experts (who were different to the advisory panel at Stage 2): media communication expert (*n* = 1), clinicians (*n* = 4), language and linguistic expert (*n* = 1); cancer survivors (*n* = 6); and the NCSM health education team (*n* = 7) with expertise in public health, nutrition, biomedical sciences, and science communication. The group of stakeholders contained several fluent speakers of each language as well as multilinguists. Stakeholders reviewed the materials in the language in which they were fluent. The designers finalized all materials after the fine-tuning exercise by stakeholders. The impact and acceptability of the campaign was evaluated as described in the study protocol through a population-based mass media campaign, which the team evaluated through prehousehold and posthousehold surveys and data from local clinics and hospitals [24].

#### Findings

The experts approved the materials as shown in the appendix. The team evaluated the impact of each campaign through comprehensive before-and-after population-based surveys. Printed outdoor advertisements required the approval of the local government council and written outdoor content had to be presented in Malay. Each private clinic had to be approached separately to enquire whether or not the clinic would allow the campaign posters and brochures to be displayed, whereas we had to approach only the district health office in order to receive approval to display materials in government clinics. In addition, local supermarkets allowed us to display campaign banners and role-ups (Table 5). Each campaign lasted 5 weeks and surveys were conducted before and after each campaign with the target population (see study protocol [24]).

**Table 5:** Comparison of campaign activities (Be Cancer Aware and Be Cancer Alert Campaign)

Be Cancer Aware (Northern Ireland)^a^	Be Cancer Alert Campaign (Malaysia)
Print materials: • 4 × Newspaper advertisements (paid), throughout October • 1 Magazine inserts × 8 women magazines, throughout October • Language: English	Print materials: • Billboards • Street buntings • Posters and brochures in public and private clinics • Roll-up banners in supermarkets • 5 × Newspaper articles (free-of-charge through media launch event) • Language: English and Malay
TV advertisements: • UTV, Channel 4, and Video on demand • Available on YouTube • Duration: 40 s; 7 weeks (2–6 ads/day) • Language: English	TV advertisements: • TV3, TV9, and 8TV • Available on YouTube • Duration: 30 s; 5 weeks (4–6 ads/day) • Language: Malay and Mandarin (TV) and English (YouTube)
Radio advertisements: • U105FM, Downtown Radio, and Classic FM • Duration: 40 s; (267 ads over 30 days) • Language: English	Radio advertisements: • Lite FM and THR Raaga • Duration: 30 s; 2 weeks (5–7 ads/day) • Language: English and Tamil
Website: https://www.becancerawareni.info/ • Paid Google search engine enhancement • Language: English	Website: http://www.becanceralert.com/ • Language: English and Malay
Social media: • 7 × Facebook advertisements • Duration: spread over 4 weeks • Language: English	Social media: • Facebook campaign, 5 weeks (4 posts/week) • Language: majority in Malay and English; few in Chinese and Tamil • Influencers: 2 posts from Malaysian influences on Facebook and Instagram Colorectal cancer: Fara Fauzana (social media influencer and moderator) and Jack Lim (radio presenter and actor) Breast cancer: Amber Chia (supermodel)
Total campaign duration: Breast cancer: 8.5 weeks (October 1–November 30, 2015)	Total campaign duration: Colorectal cancer: 5 weeks (April 2–May 6, 2018) Breast cancer: 5 weeks (September 24–October 28, 2018)

^a^Materials listed refer to the Be Cancer Aware breast cancer campaign in Northern Ireland as the Be Cancer Aware colorectal cancer campaign has not yet taken place.

## DISCUSSION

This is the first study to follow a systematic approach to develop a culturally acceptable mass media cancer awareness raising campaign for Malaysia. Local experts from the UK and Malaysia were closely involved in the design of the campaigns to ensure that there was a clear understanding of the original campaign and its suitable adaptation for Malaysians. The evaluation and reporting of the mass media campaign is nearing completion [24].

As Murray et al. highlighted, cultural adaptation is laborious and every country holds different challenges for researchers [40]. One of the main challenges we faced was that Malaysia is a multiethnic country. Feedback from the GD and EPD suggested that people were more likely to associate with, or internalize the messages of, advertisements that featured people from their own ethnic community. Therefore, the contents of the campaigns were tailored and included cancer survivors from each community. The viewing patterns of TV channels and radio stations tend to be related to ethnicity and/or language preferences. So, advertisements were created in several languages and streamed through various media channels (which posed a financial challenge in terms of funding a campaign via a research grant). Outdoor advertisements could be displayed in Malay only and locals with poor fluency in Malay may not have understood fully the cancer messages. Furthermore, presentations and discussions in public about breast health-related issues are unacceptable in a Muslim country such as Malaysia. Therefore, the campaign was limited in how it communicated the signs and symptoms of BC. It is unclear at this point in time how this restriction might affect the impact of raising awareness about BC.

In line with the majority of adapted evidence-based public health interventions, this study describes the content, context, and cultural modifications of the original BCA campaign [41]. Furthermore, the evaluation of the BCAC will include knowledge and attitude assessment, which is missing from many studies [41]. Often, research on the cultural adaptations of interventions addresses minority groups such as African Americans or Chinese Americans [8,40]. However, the adaptation of interventions that address barriers that are faced by multiethnic populations may promote cancer screening generally and not only for minority populations—a positive finding that is important for countries with diverse populations [42]. We will evaluate at a later stage the extent to which the BCAC was successful in terms of reaching and engaging a multiethnic population.

The BCACs were the first mass media breast and CRC awareness campaigns that were developed specifically for Malaysia and that followed a systematic approach. A key strength of this research was the multidisciplinary team and the close involvement of cancer survivors and health care professionals (i.e., doctors and surgeons). Previous campaigns tended to be run in isolation by one organization (e.g., by government, nongovernmental institutions, or industry) and to lack the resources for a comprehensive campaign or the rigorous evaluation of campaign effectiveness. According to Theobald et al., writing in the *Lancet* about the imperatives and opportunities in global health, building trusting partnerships with key stakeholders and the coproduction of knowledge are key to successful implementation science practice [14].

Our research provides clear evidence that it is possible to systematically develop and present a public health campaign that is designed to raise cancer awareness in an LMIC using largely a restricted or limited budget (drawn from a research grant) and in the face of other related constraints. For example, distribution of print advertisements to private clinics proved time consuming. This fact merits reflection about what might be achieved with a properly resourced budget from a government health department or a benevolent NGO. Our budget would not have afforded us the opportunity to redesign or reproduce campaign materials if the target population disliked the videos, pictures, and messages or found them unacceptable. The systematic, theoretically driven approach and thorough groundwork that was used in the adaptation and production of the materials ensured that they were context sensitive and culturally appropriate.

The team worked closely with a number of stakeholders and it is clear from the results of our coworking that the involvement of local stakeholders is key to overcoming challenges in a timely manner. Future public health efforts should include relevant key stakeholders and professionals from local communities that reflect the varied profile of the target population. Consideration of relevant sociodemographic factors in the context of conducting a cultural adaptation process (e.g., different ethnic, income, and education groups) is likely to ensure equal applicability and reach of materials and a sensitivity with respect to cultural taboos.

## CONCLUSION

The BCAC is a culturally appropriate cancer awareness raising campaign for Malaysia. The nature and extent to which it will raise awareness about cancer and encourage Malaysians to visit their doctor when they notice any abnormal changes will be evaluated as part of a large population-based study. This paper demonstrates the benefits of adopting a systematic and theoretically driven approach to the cultural adaptation of best available evidenced public health interventions. The strategies and challenges that are presented in this paper provide lessons and guidance for public health officers and NGOs about the planning and conduct of future campaigns in Malaysia as well as the design of public health campaigns in other South East Asian countries.

## SUPPLEMENTARY MATERIAL

Supplementary material is available at *Translational Behavioral Medicine* online.

**Funding:** This study is funded by the UK Medical Research Council—Newton Ungku Omar Funding.

## Compliance with Ethical Standards

**Conflicts of Interest:** The authors declare that they have no conflicts of interest.

**Authors’ Contributions:** MDo and TTS conceptualized and planned the project and are the Co-PIs and overseers of the successful grant award from UK MRC-Newton Ungku Omar Fund. TTS, MDa, LSY, DS and DP planned and coordinated the study. DP conducted the group discussions and TTS conducted the expert panel discussions. DS and DP analyzed the group discussions. DS drafted the manuscript. All authors reviewed and approved the final manuscript.

**Ethical Approval:** All procedures performed in studies involving human participants were in accordance with the ethical standards of Medical Research Ethics Committee, University Malaya Medical Centre (ID: 2016126–4668) and the National Medical Research Register (ID: NMRR-17-2788-35,613 and NMRR-18-1961-42562) and with the 1964 Helsinki declaration and its later amendments or comparable ethical standards. This article does not contain any studies with animals performed by any of the authors.

**Informed Consent:** Informed consent was obtained from all individual participants included in the study.

## Supplementary Material

ibz134_suppl_Supplementary_Material_1

ibz134_suppl_Supplementary_Material_2
