# Supplementary material for: The systematic cultural adaptation of a UK public health cancer awareness raising programme for Malaysia: the Be Cancer Alert Campaign
Source: Transl Behav Med. 2019 Oct 4;9(6):1087–99. doi: 10.1093/tbm/ibz134 (PMC6875648; doi:10.1093/tbm/ibz134)
Supplement: ibz134_suppl_Supplementary_Material_2 [file ibz134_suppl_supplementary_material_2.docx]

**Supplement 2**


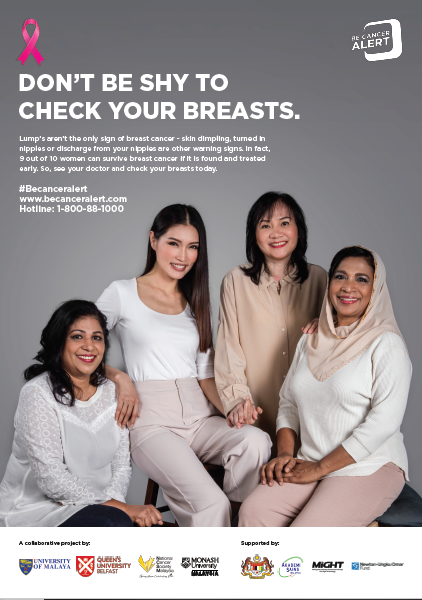


**Picture 1** Breast cancer print campaign material


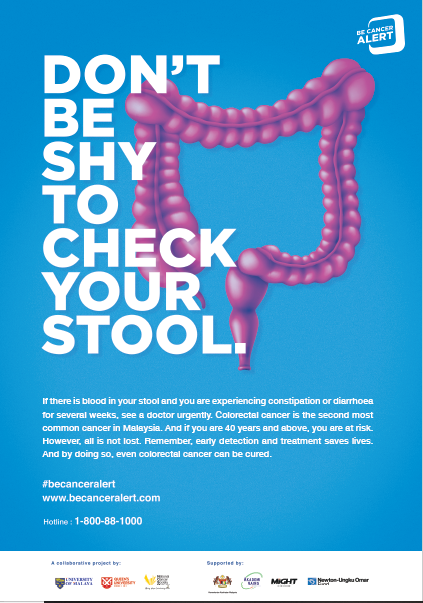


**Picture 2** Colorectal cancer print campaign material
